# The potential of sperm bovine protamine as a protein marker of semen production and quality at the National Artificial Insemination Center of Indonesia

**DOI:** 10.14202/vetworld.2021.2473-2481

**Published:** 2021-09-23

**Authors:** Berlin Pandapotan Pardede, Tulus Maulana, Ekayanti Mulyawati Kaiin, Muhammad Agil, Ni Wayan Kurniani Karja, Cece Sumantri, Iman Supriatna

**Affiliations:** 1 Reproductive Biology Study Program, Faculty of Veterinary Medicine, IPB University, Dramaga, Bogor 16680, Indonesia; 2 Animal Reproduction Biotechnology Research Group, Research Center for Biotechnology, Indonesian Institute of Sciences, West Java, Indonesia; 3 Department of Veterinary Clinic, Reproduction, and Pathology, Faculty of Veterinary Medicine, IPB University, Dramaga, Bogor 16680, Indonesia; 4 Department of Animal Production and Technology, Faculty of Animal Science, IPB University, Dramaga, Bogor 16680, Indonesia.

**Keywords:** bull, protamine, protein marker, semen production, semen quality

## Abstract

**Background and Aim:**

Protamine (PRM) is the major protein in the sperm nucleus and plays an essential role in its normal function. Moreover, PRM has great potential as a protein marker of semen production and quality. This study aimed to assess the potential of sperm bovine PRM as a protein marker of semen production and quality in bulls at the National Artificial Insemination (AI) Center of Indonesia.

**Materials and Methods:**

The semen production capacity of each bull was collected from frozen semen production data at the Singosari AI Center for 6 months, and was then divided into two groups (high and low). A total of 440 frozen semen straws from six Limousin (LIM), six Friesian Holstein (FH), six Peranakan Ongole (PO), and four Aceh bulls aged 4-5 years were used in the study. The frozen semen was used to measure the concentration of PRM1, PRM2, and PRM3 using the enzyme immunoassay method. The frozen semen was also used to assess the quality of the semen, including progressive motility (PM) through computer-assisted semen analysis, sperm viability through eosin–nigrosin analysis, and the DNA fragmentation index through Acridine Orange staining.

**Results:**

PRM1 was significantly higher in all bull breeds included in the study (p<0.00), followed by PRM2 (p<0.00) and PRM3 (p<0.00). PRM1 significantly affected semen production in LIM, FH, PO, and Aceh bulls (p<0.05). Moreover, PRM2 significantly affected semen production only in FH and Aceh bulls (p<0.05), whereas PRM3 affected this parameter in PO and Aceh bulls exclusively (p<0.05). Consistently and significantly, PRM1 was positively correlated with the PM and viability of sperm and negatively associated with its DNA fragmentation in LIM, FH, PO, and Aceh bulls (p<0.05; p<0.01). The correlation analysis between PRM2 and PRM3 and semen quality parameters varied across all bull breeds; some were positively and negatively correlated (p<0.05; p<0.01), and some were not correlated at all.

**Conclusion:**

PRM1 has excellent potential as a protein marker of semen production and quality in bulls at the National AI Center of Indonesia.

## Introduction

Bull fertility is related to semen quality [1,2]; the semen must contain good-quality sperm to fertilize oocytes until conception occurs [3]. Mishra *et al*. [4] reported that the classical semen parameters were currently considered insufficient to predict a bull’s fertility. Therefore, genetic markers for predicting fertility rates more accurately are needed and can be helpful for the selection of bulls and the improvement of subsequent cattle populations [4]. The use of genes and proteins in sperm and seminal plasma combined with semen quality evaluation as molecular markers has been widely reported and is considered more effective [5-8]. However, using a combination of molecular markers and semen quality will be very beneficial and have a significant impact on economic aspects, particularly regarding the selection and raising of bulls for the Artificial Insemination (AI) program. Moreover, using molecular markers as the fertility selection parameter of a bull could increase the time and cost-efficiency of keeping the bull, because the fertility selection could be achieved as early as possible, when molecular markers determine the quality and production of semen.

Protamine (PRM) is the major protein in the sperm nucleus and plays an essential role in its normal function, including the DNA-binding process [8]. PRM is formed during the spermiogenesis phase [9], during which a protein-replacement process occurs in the sperm nucleus. Histone proteins that initially dominate the sperm nucleus are then replaced by PRM through complex processes, such as methylation, phosphorylation, and ubiquitination [8]. PRM will pack sperm DNA optimally to increase chromatin condensation, which will protect the genetic integrity of the paternal genome against nuclease enzymes, mutagens, and other factors that can damage DNA [10]. Sperm PRM differs among species; in humans [11], mice, rats, and hamsters [12], two types of PRM, namely, PRM1 and PRM2, play a role in the normal function of sperm. Beletti *et al*. [13] reported that only one type of PRM, namely, PRM1, plays a dominant role in the normal function of bull sperm. However, Ferraz *et al*. [14] reported that PRM1, PRM2, and PRM 3 are expressed in bovine. In pigs, PRM1 is a type of PRM that plays a role in the normal function of sperm because of the *PRM2* gene mutase [15].

Various effects of deficiency of PRM on semen quality have been reported, such as increased morphological abnormalities and DNA damage, acrosome and membrane defects, and immotile sperm in mice [16,17]. Several negative effects of PRM deficiency on human sperm quality and fertility have also been reported (sperm decreased motility, concentration, and DNA damage) [18]. In addition, it can also cause DNA damage in boars [19], reduced semen quality (e.g., motility, viability, and the integrity of membranes) in canine [20], and reduce semen quality (e.g., volume, concentration, viability, plasma membrane, and DNA damage) in bulls [21,22]. Various studies related to the molecular research on the sperm forms of PRM (PRM1, PRM2, and PRM3) in bulls and their relation to semen quality and fertility have been reported [1,3,14,22-24].

However, studies of bovine PRM sperm in bulls in Indonesia have not been published. Hence, this study was carried out to assess PRM1, PRM2, and PRM3 in bull sperm, as well as their relationship with semen production and quality, which is expected to prove the potential of bovine PRM as a biomarker of semen production and quality.

## Materials and Methods

### Ethical approval

The frozen semen used in this study was from the Singosari AI Center. Starting from the management of bulls, every procedure (i.e., the collection of fresh semen and its freezing until ready to be marketed) was in accordance with Indonesia’s operational standards, namely, SNI ISO 9001: 2015 No. G.01-ID0139-VIII-2019, and was supervised by a veterinarian. Each stage of this study considered every aspect of animal welfare and met the requirements for ethical clearance by the Animal Care and Uses Committee.

### Study period, location, and sample collection

The research was conducted from December 2020 to February 2021 at the Laboratory of Animal Reproduction, Breeding and Cell Culture, Research Center for Biotechnology, Indonesian Institute of Sciences, West Java, Indonesia; and the Laboratory of Microbiology and Immunology, Primate Research Center, IPB University, West Java, Indonesia. Frozen semen (330 straws for analysis of bovine PRM; 110 straws for semen quality analysis) from six Limousin (LIM), six Friesian Holstein (FH), six Peranakan Ongole (PO), and four Aceh bulls aged 4-5 years were used in this study.

### Semen production analysis

The semen production capacity of each bull was obtained from frozen semen production data at the Singosari AI Center±over a period of approximately 6 months. Each bull was classified into one of two groups (high and low) based on the frozen semen production capacity (straw production per collection) (Table-1).

**Table-1:** Semen production capacity (straw production per collection) in Limousin, Friesian Holstein, Peranakan Ongole, and Aceh bulls.

Breed	Variables	Semen production groups (mean±SEM)			

		High	n	Low	n
Limousin	Straw production per collection (pieces)	484.31±12.58^a^	141	303.75±12.72^b^	101
Friesian Holstein	Straw production per collection (pieces)	602.89±21.77^a^	74	225.19±21.97^b^	37
Peranakan Ongole	Straw production per collection (pieces)	328.49±10.84^a^	138	232.43±10.83^b^	99
Aceh	Straw production per collection (pieces)	272.33±18.98^a^	21	210.79±16.69^b^	29

n=number of ejaculates

### Measurement of bovine PRM1

The concentration of PRM1 in each bull used in this study was measured using a bovine PRM1 ELISA kit (Cat No. MBS2609702, MyBioSource.com), according to the manufacturer’s instructions. Frozen semen from each bull was thawed for 30 s in a water bath at 37°C. A total of 100 mL of semen was centrifuged at 3000 rpm for 15 min and washed with phosphate-buffered saline (PBS) twice. Subsequently, the sperm was tested by ELISA according to the manufacturer’s instructions. Briefly, the reagents, samples, and standards were prepared. The sample and the standard bovine PRM1 sample were then added to the corresponding reaction wells, which were covered with adhesive tape and incubated at 37°C for 90 min. The biotinylated anti-bovine PRM1 antibody solution was prepared 30 min before experimentation. The ELISA plate was washed twice and the antibody solution was added to the wells (100 mL), which were sealed with adhesive tape and incubated at 37°C for 60 min. The ELISA plate was washed 3 times and the enzyme-conjugated solution (100 mL) that was prepared 30 min earlier was added to the wells, which were sealed and incubated at 37°C for 30 min. Subsequently, the ELISA plate was washed 5 times, the Color Reagent solution (100 mL) was added, and the plate was placed in a dark incubator at 37°C for 30 min. In the final step, 100 mL of Color Reagent C was added and mixed. Finally, the absorbance was measured at 450 nm using an automatic plate reader (ELISA reader) within 10 min.

### Measurement of bovine PRM2

Frozen semen from each bull was thawed for 30 s in a water bath at 37°C. A total of 100 mL of thawed semen was centrifuged at 3000 rpm for 15 min, washed with a solution of PBS twice, and used to measure the concentration of PRM2 using a bovine PRM2 ELISA kit (Paint No. MBS9712914, MyBioSource.com). Briefly, all the required reagents were prepared. First, 50 μL of the standard diluent was added to the standard well. Next, 40 μL of sample diluent and 10 μL of sample were added to the wells, and the plate was covered and incubated at 37°C for 45 min. Each well was aspirated and washed with a wash buffer (250 mL), and the process was repeated 4 times, for a total of five washes (1-3 min each time). Subsequently, 50 mL of HRP-conjugated detection antibody was added to each well, and the plate was covered and incubated at 37°C for 30 min. The ELISA plate was then aspirated and washed 5 times, followed by the addition of 50 mL each of chromogen solutions A and B to each well. The plate was incubated at 37°C for 15 min in the dark. Finally, 50 mL of Stop Solution was added to each well and the optical density (OD) was read at 450 nm using a microtiter plate reader within 15 min of the chromogenic reaction.

### Measurement of bovine PRM3

The semen samples employed for PRM3 measurements using a bovine PRM3 ELISA (Cat No. MBS9392614, MyBioSource.com) were thawed, centrifuged, and washed as described for the PRM1 and PRM2 concentration measurements. Frozen semen from each bull was thawed for 30 s in a water bath at 37°C. Next, 50 mL of the standard Bovine PRM3 sample was added to the appropriate standard well and 50 μL of the sample was added to each sample well. Subsequently, 100 μL of the HRP-conjugated Reagent was added to each well, and the plate was covered with a Closure Plate Membrane and incubated at 37°C for 60 min. All wells were washed 4 times and 50 μL of Chromogen Solution A was added to each well, followed by the addition of 50 μL of Chromogen Solution B to each well in the dark. The resulting solution was mixed gently and the plate was incubated at 37°C for 15 min in the dark. Finally, 50 μL of Stop Solution was added to each well and the OD was measured at 450 nm using an ELISA reader within 15 min of the reaction.

### Semen quality assessments

Frozen semen from each bull was thawed for 30 s in a water bath at 37°C. The semen was removed from the straw and placed in a microtube. During the evaluation, the semen was stored on a warm stage at 37°C. Sperm progressive motility (PM%) was analyzed using a computer-assisted semen analysis based on Sundararaman *et al*. [25]. A total of 10 μL was dropped onto a glass slide and covered with a coverslip and observed using the Sperm Vision Program (Minitüb, Tiefenbach, Germany). Specific settings for bovine sperm were used to evaluate a total of 200-750 sperm cells in four fields. Eosin–nigrosin staining (0.2 g of eosin, 2 g of nigrosin, mixed with 100 μL of distilled water) was used to evaluate sperm viability. A total of 10 μL of semen was dropped onto a glass object and combined with the eosin–nigrosin solution. A smear of the sample was dried using a heating table and then observed under a light microscope at 40×. A total of 200 sperm cells were observed and counted; the dead sperm were stained red, and the living sperm were not colored (transparent) [26].

The DNA fragmentation index (DFI%) was analyzed using the acridine orange (AO) assay based on the method of Esteves *et al*. [27]. First, a smear of 5-10 μL of semen was fixed with Carnoy’s solution for 2 h. Subsequently, the samples were stained with the AO solution for 5 min in a dark room and washed with distilled water, then covered with a coverslip and examined under a Zeiss AxioPhot fluorescence microscope at an excitation wavelength of 450-490 nm. A total of 500 sperm cells were observed and counted; sperm cells with normal DNA integrity were colored with green fluorescence, whereas sperm with DNA fragmentation was colored with yellow–orange-to-red fluorescence.

### Statistical analysis

Semen production capacity data and the effect of the PRM1, PRM2, and PRM3 proteins in the high and low production groups in each breed of bulls were analyzed using Student’s t-test. Data on the comparison of the PRM1, PRM2, and PRM3 proteins in each breed of bulls were analyzed using analysis of variance, and Duncan’s multiple range tests were used as a further test if a significant difference was found. The relationship between the PRM1, PRM2, PRM3 proteins, and semen quality was analyzed using Spearman’s Rho correlation test. All data analyses performed in this study were processed using SPSS ver. 25.0 (IBM, Armonk, NY, USA). Data are presented as the mean±standard error of the mean (SEM).

## Results

**Figure-1:**
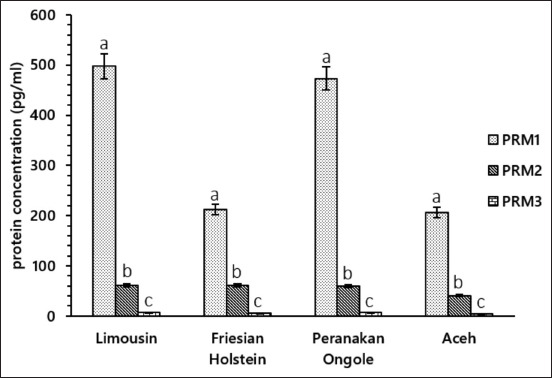
Bovine Protamine 1 (PRM1) exhibited the highest concentration (p<0.00) in the sperm of Limousin, Friesian Holsten, Peranakan Ongole, and Aceh bulls, followed by PRM2 (p<0.00) and PRM3 (p<0.00).

PRM1, PRM2, and PRM3 were detected and measured in all bulls used in this study (Figure-1). The average PRM1 concentration detected here was 497.72±62.41 (LIM), 211.77±15.37 (FH), 473.16±75.19 (PO), and 206.25±10.09 (Aceh) pg/mL. The PRM2 concentration was 60.95±2.22 (LIM), 61.49±5.61 (FH), 59.96±4.44 (PO), and 40.72±5.34 (Aceh) pg/mL. The PRM3 concentration was 6.92±0.17 (LIM), 5.04±0.36 (FH), 7.04±0.27 (PO), and 4.48±0.44 (Aceh) pg/mL. The level of PRM1 was significantly higher in all bull breeds included in the study (p<0.00), followed by PRM2 (p<0.00) and PRM3 (p<0.00). These results were then used to analyze further the relationship between bovine PRM levels and semen production and quality. The production of semen in LIM, FH, PO, and Aceh bulls was significantly higher (484.31±12.58, 602.89±21.77, 328.49±10.84, and 272.33±18.98 straws per ejaculate; p<0.05) in the high production group compared with the low production group (303.75±12.72, 225.19±21.97, 232.43±10.83, and 210.79±16.69 straws per ejaculate) (Table-1).

**Figure-2:**
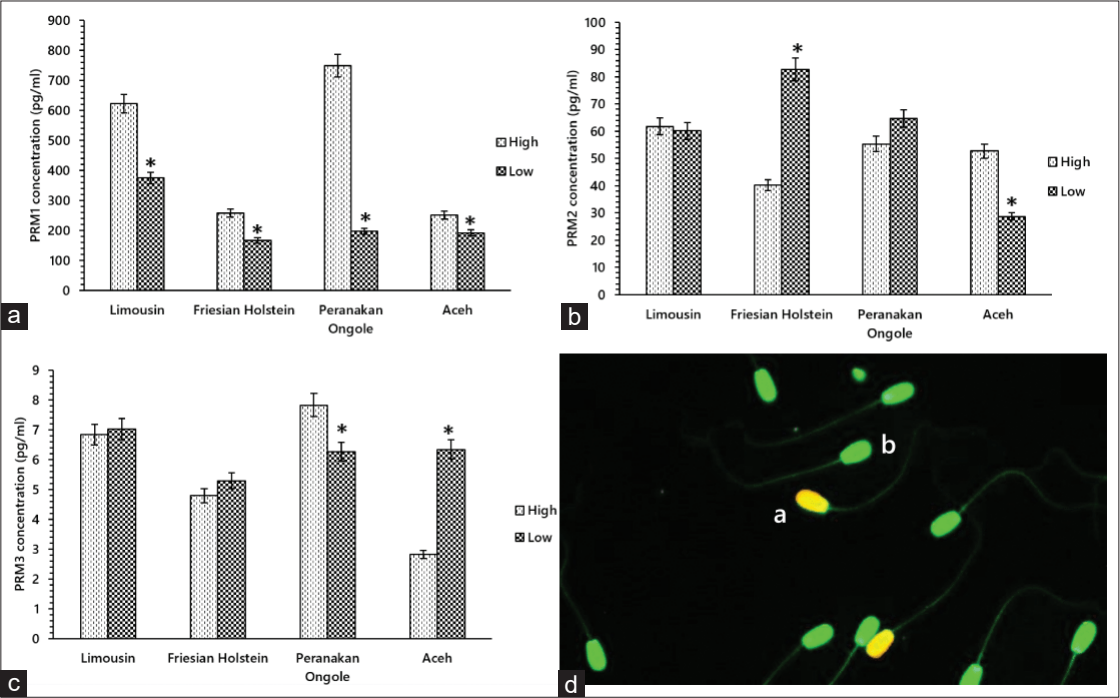
Relationship between the Protamine (PRM1), PRM2, and PRM3 concentrations and frozen semen production capacity in Limousin, Friesian Holstein (FH), Peranakan Ongole (PO), and Aceh bulls (a-c). The PRM1 concentrations were higher (p<0.05) in the high production groups in Limousin, FH, PO, and Aceh bulls (a). The concentrations of PRM2 and PRM3 varied among the production groups in all bull breeds (b and c). Results of DNA fragmentation staining in sperm using acridine orange (d); sperm with DNA fragmentation exhibited yellow-orange fluorescence (a), and normal sperm showed green fluorescence (b).

PRM1 significantly affected semen production in LIM (high, 621.44±73.12 vs. low, 374.00±85.90 pg/mL), FH (high, 257.11±18.09 vs. low, 166.44±12.76 pg/mL), PO (high, 749.22±66.43 vs. low, 197.11±23.73 pg/mL), and Aceh (high, 270.33±18.98 vs. low, 210.79±16.68 pg/mL) bulls (p<0.05) (Figure-2). The levels of PRM2 were significantly higher in the low production groups of FH (high, 40.27±1.97 vs. low, 82.72±4.17 pg/mL) bulls, and significantly lower in the low production groups of Aceh (high, 25.29±0.84 vs. low, 56.16±8.17 pg/mL) bulls (p<0.05). There was no difference in PRM2 between the high and low production groups in LIM (61.78±3.03 vs. 60.12±3.42 pg/mL) and PO (55.37±6.97 vs. 64.54±5.45 pg/mL) bulls (Figure-2). PO (7.82±0.34 vs. 6.26±0.20 pg/mL) bulls showed a higher PRM3 level in the high production group (p<0.05). Aceh (2.82±0.35 vs. 6.34±0.18 pg/mL) bulls showed a higher PRM3 level in the low production group (p<0.05); however, there was no significant difference (p>0.05) in LIM (6.83±0.19 vs. 7.01±0.30 pg/mL), and FH (4.79±0.55 vs. 5.29±0.49 pg/mL) bulls (Figure-2).

**Figure-3:**
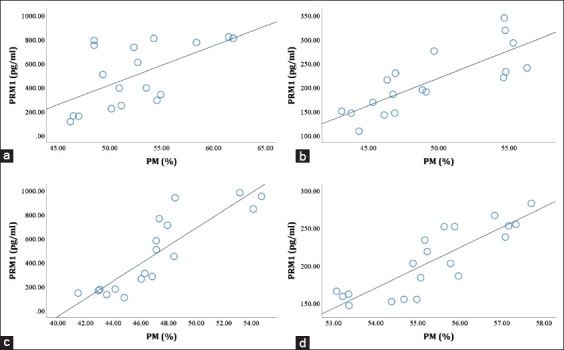
Relationship between Protamine 1 (PRM1) (pg/mL) and PM (%) in Limousin (r=0.603) (p<0.05) (a), FH (r=0.846) (p<0.01) (b), Peranakan Ongole (r=0.920) (p<0.01) (c), and Aceh (r=0.851) (p<0.01) (d) bulls.

**Figure-4:**
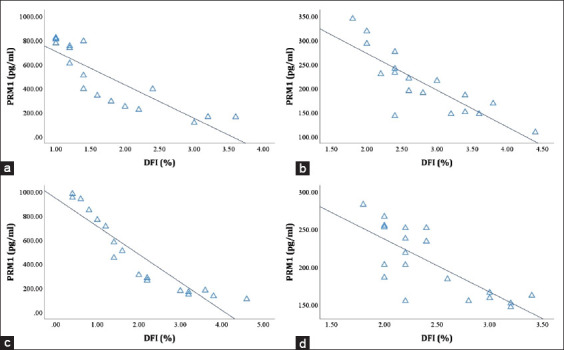
Relationship between Protamine 1 (%) and DNA fragmentation index (%) in Limousin (r=−0.932) (a), Friesian Holstein (r=−0.824) (b), Peranakan Ongole (r=−0.982) (c), and Aceh (r=−0.761) (p<0.01) (d) bulls.

Consistently and significantly, PRM1 was positively correlated with the PM (Figure-3) and viability (Table-2) of sperm, and negatively associated with DNA fragmentation (Figure-4) in LIM, FH, PO, and Aceh bulls (p<0.05; p<0.01). The analysis of the correlation between PRM2 and PRM3 and semen quality parameters revealed that it varied across all bull breeds; some were positively and negatively correlated (p<0.05; p<0.01), and some were not correlated at all (Table-2).

**Table-2:** Correlation between PRM1, PRM2, and PRM3 and semen quality parameters in Limousine, FH, PO, and Aceh bulls.

Breed	Sperm protamine	Type of correlation coefficient	Correlation coefficient	p-value
LIM	PRM1 versus viability (%)	Spearman’s Rho	0.625	0.006**
	PRM2 versus PM (%)	Spearman’s Rho	-0.288	0.247
	PRM2 versus viability (%)	Spearman’s Rho	-0.193	0.442
	PRM2 versus DFI (%)	Spearman’s Rho	0.264	0.291
	PRM3 versus PM (%)	Spearman’s Rho	-0.314	0.205
	PRM3 versus viability (%)	Spearman’s Rho	-0.018	0.945
	PRM3 versus DFI (%)	Spearman’s Rho	0.158	0.531
FH	PRM1 versus viability (%)	Spearman’s Rho	0.740	0.000**
	PRM2 versus PM (%)	Spearman’s Rho	-0.713	0.000**
	PRM2 versus viability (%)	Spearman’s Rho	-0.599	0.003**
	PRM2 versus DFI (%)	Spearman’s Rho	0.610	0.002**
	PRM3 versus PM (%)	Spearman’s Rho	0.001	0.997
	PRM3 versus viability (%)	Spearman’s Rho	0.206	0.413
	PRM3 versus DFI (%)	Spearman’s Rho	0.089	0.724
PO	PRM1 versus viability (%)	Spearman’s Rho	0.819	0.000**
	PRM2 versus PM (%)	Spearman’s Rho	-0.003	0.990
	PRM2 versus viability (%)	Spearman’s Rho	0.070	0.781
	PRM2 versus DFI (%)	Spearman’s Rho	0.180	0.475
	PRM3 versus PM (%)	Spearman’s Rho	0.820	0.000**
	PRM3 versus viability (%)	Spearman’s Rho	0.684	0.002**
	PRM3 versus DFI (%)	Spearman’s Rho	-0.673	0.002**
ACEH	PRM1 versus viability (%)	Spearman’s Rho	0.469	0.037*
	PRM2 versus PM (%)	Spearman’s Rho	0.324	0.163
	PRM2 versus viability (%)	Spearman’s Rho	-0.132	0.2579
	PRM2 versus DFI (%)	Spearman’s Rho	-0.219	0.354
	PRM3 versus PM (%)	Spearman’s Rho	-0.589	0.006**
	PRM3 versus viability (%)	Spearman’s Rho	-0.262	0.265
	PRM3 versus DFI (%)	Spearman’s Rho	0.429	0.059

** Correlation is significant at the 0.01 level;

* correlation is significant at the 0.05 level

## Discussion

PRM is the major protein in the sperm nucleus and is formed during the spermiogenesis phase [9]. Overall, in this study, PRM1 was the PRM type with the highest concentration (p<0.00) compared with other PRM types in the sperm of all bull breeds. Ferraz *et al*. [14] reported similar results, that is, PRM1 was the predominant type of PRM and had the highest amount compared with PRM2 and PRM3 in bovine testes. Ganguly *et al*. [22] also reported that, in the sperm of Frieswal crossbred bulls, the level of PRM1was higher than that of PRM2. The previous comparisons between PRM1, PRM2, and PRM3 in LIM, FH, PO, and Aceh breeds have not been reported; therefore, our results, which were obtained using mainly the protein approach, provide new information on PRM concentrations in these bull breeds. The identification of the *PRM1* gene in Aceh bulls at the DNA level was reported previously by Helmi *et al*. [28]. The *PRM1* gene of Aceh bulls is similar to the *PRM1* gene in *Bos taurus* and *Bos indicus*. In this study, at the protein level, PRM1 had the highest concentration compared with PRM2 and PRM3, although its concentration varied according to breed, overall.

The PRM1 concentration in the high frozen semen production group in LIM, FH, PO, and Aceh bulls was significantly different from the low production group (p<0.05). Ismaya [29] stated that bull semen production may be affected by various factors, such as age, genetics, temperature, season, frequency of ejaculation, feed, and body weight. Therefore, the PRM1 concentrations detected in sperm in this study may include genetic factors that influence the high and low semen production detected in LIM, FH, PO, and Aceh bulls (Figure-2). Suyadi *et al*. [30] stated that semen volume, the number of sperm, concentration, and sperm motility affect frozen semen production in bulls. However, abnormal PRM expression will decrease the number, concentration, and motility of sperm [31]. Pool *et al*. [32] also added that PRM deficiencies in sperm can cause testicular disorders in ram and lead to decreased semen production and concentration and a diminished number of sperm per ejaculate. Therefore, it is suggested that the PRM1 concentration detected in this study plays an essential role in semen production in bulls and has the potential as a biomarker of semen production in bulls.

The PRM2 and PRM3 concentrations in all bull breeds varied from one another (Figure-1), as did their relationship to semen production (Figure-2) and semen quality (Table-2). Studies related to the relationship between PRM2 and PRM3 and semen production and quality, especially in bulls, are limited. Many questions remain unanswered regarding its function, expression, regulation, and phylogenetic distribution [33]. Kumar *et al*. [34] reported that PRM3 did not affect good and poor semen quality, such as volume, concentration, number of sperm per ejaculate, and sperm motility. A decrease in sperm motility due to the absence of PRM3 in mice has been informed by Grzmil *et al*. [33]. Lv *et al*. [35] reported that PRM2 and PRM3 in Yanbian Yellow bulls play an essential role in sperm motility because of severe membrane damage to sperm. Schneider *et al*. [16] also added that severe membrane damage in the sperm of mice with PRM2 deficiency could also result in histone damage and impaired DNA hyper-condensation.

In contrast to the results of Lv *et al*. [35], PRM2 did not affect sperm quality, including motility, in crossbred Frieswal bulls [22]. Here, a decrease in PRM3 concentration followed by a decline in PM, sperm viability, and an increase in DNA damage was found in PO bulls (p<0.01), but not in LIM, FH, and Aceh bulls. Variations in the concentration of the PRM2 and PRM3 proteins in bulls can be caused by various factors, including the environment and the presence of gene mutases [8]. Vihinen [36] also revealed that the levels of original genetic variations in DNA and RNA could affect the final protein product, causing variable protein concentrations. These protein variations have many diverse effects that can affect sequence, form, establishment, interactions, regulation, profusion, and other traits [36]. Grzmil *et al*. [33] also stated that sperm’s normal function pertaining to each sperm quality parameter is not controlled by one gene or protein; rather, it is modulated by more than one molecule in a complex process. Therefore, the existence of a disturbance in each gene or protein encoding the parameters of sperm quality will have an impact on bull fertility.

Sperm motility is an essential characteristic for the ability to fertilize [37]. The Indonesian National Standard number 4869.1-2017 regarding frozen semen for bulls [38] requires that post-thawing frozen semen is at least at a minimum value of 40%. Garner and Hafez [39] stated that motility is one of the parameters of sperm quality that is crucial for sperm to pass through the cervix; even PM helps sperm penetrate the cumulus oophorous and the pellucid zone. Pardede *et al*. [26] also added that the PM of sperm is very closely correlated with the conception rate of cows. In this study, overall, the PM of the sperm from all bulls met these requirements, that is, it was above 40% (Figure-3). PRM1 was significantly positively correlated with the PM of sperm (p<0.05; p<0.01) in all bulls; thus, PRM1 seemed to play a role in the PM of sperm. The reduction of sperm motility by decreased concentrations of the PRM1 protein has been reported previously [22,31,32,40]. Schneider *et al*. [16] reported that the concentration of Ca^2+^ as a control for PRM phosphorylation was decreased in sperm with PRM deficiency, causing a decrease in the quality of the sperm plasma membrane, motility, and DNA. Miyagawa *et al*. [41] reported a correlation between increased DNA fragmentation and abnormalities in the sperm tail midpiece, which contains mitochondria. The increased denaturability of DNA stimulates the apoptotic signaling pathway, affects mitochondria, and decreases motility. Moreover, Takeda *et al*. [17] reported that damage to the mitochondrial membrane, which is vital for flagella movement and sperm motility, was more significant in PRM deficiency conditions than in normal mice.

Sperm viability testing was carried out to test for damage to the sperm membrane [42]. Living sperm have a suitable membrane; thus, the eosin–nigrosin dye does not enter these cells. In contrast, dead sperm has a damaged and leaky membrane that is nonfunctional, which causes the dye to penetrate the cells, and the color of the head becomes purple-red [26]. In this study, the concentration of PRM1 in sperm affected the quality of the sperm membrane (p<0.05; p<0.01) in all bull breeds. A similar result was reported by Schneider *et al*. [16], who found that membrane damage occurred as a result of PRM deficiency. Damage to the membrane will lead to various damages to sperm, including damage to the acrosome and DNA chromatin. However, sperm DNA is the sperm component most affected if there is an abnormal expression or deficiency of PRM1 in bulls [8]. As the major protein in the sperm nucleus of bulls, PRM1, like arginine, plays an essential role in the paternal genome condensation. This DNA–PRM bond will produce a sperm nucleus that is denser and more hydrokinetic [43,44]. Sperm with a hydrokinetic nucleus is indispensable in the fertilization process, in which the sperm will move quickly and be able to fertilize oocytes [10]. Therefore, it is not surprising that PRM1 was correlated with the DFI% (p<0.01) in all bulls in this study (Figure-4). Decreased expression or deficiency of PRM, which causes increased DNA damage, has been reported in many species, such as mice [17], boars [19,45], canines [46], humans [18], and bovine [1,21,47]. Dogan *et al*. [1] and Pardede *et al*. [26] stated that DNA fragmentation would decrease fertility, as observed in the low conception rate in cows inseminated with sperm with this type of damage.

Moreover, sperm DNA damage will inhibit embryo development, reduce implantation ability, and result in pregnancy failure [48,49]. Bochenek *et al*. [50] reported a decrease in fertility in bulls with sperm DNA damage greater than 10%. Overall, DNA damage in this study was less than 5% and was still within normal limits. It must be considered that each parameter of semen quality plays its role until fertilization occurs. Overall, it is suggested that PRM1 plays an essential role in controlling the quality of semen, which will impact the fertility of bulls. However, this study provides new information regarding the regulation afforded by, and the important function of bovine PRM in bulls in Indonesia. Further and more complex studies at the molecular level are necessary, including at the DNA and RNA levels, especially regarding PRM2 and PRM3 in bulls.

## Conclusion

PRM1 has excellent potential as a protein marker of semen production and quality in bulls at the National AI Center of Indonesia.

## Authors’ Contributions

BPP, MA, IS, NWKK, and CS: Conceptualized and designed this study. BPP: Performed the experiment under the guidance of TM and EMK. BPP: Analyzed the results, literature search, and wrote the first manuscript draft. MA, IS, NWKK, and CS: Edited, and revised the final manuscript. All authors read and approved the final manuscript.

## References

[1] Dogan S, Vargovic P, Oliveira R, Belser L.E, Kaya A, Moura A, Sutovsky P, Parrish J, Topper E, Memili E (2015). Sperm protamine-status correlates to the fertility of breeding bulls. Biol. Reprod.

[2] Kebede A (2018). Review on factors affecting success of artificial insemination. Int. J. Curr. Res. Aca. Rev.

[3] Hamilton T.R.S, Simoes R, Mendes C.M, Goissis M.D, Nakajima E, Martins E.A.L, Vicintin J.A, Assumpcao M.E.O (2019). Detection of protamine 2 in bovine spermatozoa and testicles. Andrology.

[4] Mishra C, Palai T.K, Sarangi L.N, Prusty B.R, Maharana B.R (2013). Candidate gene markers for sperm quality and fertility in bulls. Vet. World.

[5] De Oliveira R.V, Dogan S, Belser L.E, Kaya A, Topper E, Moura A, Thibaudeau G, Memili E (2013). Molecular morphology and function of bull spermatozoa linked to histones and associated with fertility. Reproduction.

[6] Bromfield J.J (2016). A role for seminal plasma in modulating pregnancy outcomes in domestic species. Reproduction.

[7] Westfalewicz B, Dietrich M.A, Mostek A, Partyka A, Bielas W, Nizanski W, Ciereszko A (2017). Identification and functional analysis of bull (*Bos taurus)* cauda epididymal fluid proteome. J. Dairy. Sci.

[8] Pardede B.P, Supriatna I, Agil M (2020). Protamine and other proteins in sperm and seminal plasma as molecular markers of bull fertility. Vet. World.

[9] Depa-Martynow M, Kempisty B, Jagodzinski P.P, Pawelzyk L, Jedrzejczak P (2011). Impact of protamine transcripts and their proteins on the quality and fertilization ability of sperm and the development of preimplantation embryos. Reprod. Biol.

[10] Oliva R (2006). Protamines and male infertility. Hum. Reprod. Update.

[11] Balhorn R, Steger K, Bergmann M, Schuppe H.C, Neuhauser S, Balhorn M.C (2018). New monoclonal antibodies specific for mammalian protamines P1 and P2. Syst. Biol. Reprod. Med.

[12] Bower P.A, Yelick P.C, Hecht N.B (1987). Both P1 and P2 protamine genes are expressed in mouse, hamster, and rat. Biol. Reprod.

[13] Beletti M.E, Costa L.F, Guardieiro M.M (2005). Morphometric features and chromatin condensation abnormalities evaluated by toluidine blue staining in bull spermatozoa. Braz. J. Morphol. Sci.

[14] Ferraz M.A.M, Simoes R, Barros F.O, Millazzoto. M.P, Visintin J.A, Assumpcao M.E.O (2013). Gene expression profile of protamines and transition nuclear proteins in bovine testis. Braz. J. Vet. Res. Anim. Sci.

[15] Maier W.M, Nussbaum G, Domenjoud L, Klemm U, Engel W (1990). The lack of protamine 2 (P2) in boar and bull spermatozoa is due to mutations within the P2 gene. Nucleic Acids Res.

[16] Schneider S, Balbach M, Jikeli J.F, Fietz D, Nettersheim D, Jostes S, Schmidt R, Kressin M, Bergmann M, Wachten D, Steger K, Schorle H (2016). Re-visiting the protamine-2 locus:Deletion, but not haploinsufciency, renders male mice infertile. Sci. Rep.

[17] Takeda N, Yoshinaga K, Furushima K, Takamune K, Li Z, Abe S, Aizawa S, Yamamura K (2016). Viable offspring obtained from Prm1-deficient sperm in mice. Sci. Rep.

[18] Amor H, Zeyad A, Bakry M.S, Bosilah A.M.H, Ali H.B, Hammadeh M.E (2018). Protamine ratio as predictor of the fertility potential of sperm by couple undergoing ICSI. Int. J. Women's Health Reprod.

[19] Khezri A, Narud B, Stenseth E, Johannisson A, Myromslien F.D, Gaustad A.H, Wilson R.C, Lyle R, Morrell J.M, Kommisrud E.K, Ahmad R (2019). DNA methylation patterns vary in boar sperm cells with different levels of DNA fragmentation. BMC Genomics.

[20] Qamar A.Y, Fang X, Kim M.J, Cho J (2019). Myoinositol supplementation of freezing medium improves the quality-related parameters of dog sperm. Animals.

[21] Fortes M.R.S, Satake N, Corbet D.H, Corbet N.J, Burns B.M, Moore S.S, Boe-Hansen G.B (2014). Sperm protamine deﬁciency correlates with sperm DNA damage in *Bos indicus* bulls. Andrology.

[22] Ganguly I, Gaur G.K, Kumar S, Mandal D.K, Kumar M, Singh U, Kumar S, Sharma A (2012). Differential expression of protamine 1 and 2 genes in mature spermatozoa of normal and motility impaired semen producing crossbred Frieswal (HF x Sahiwal) bulls. Res. Vet. Sci.

[23] Feugang J.M, Rodriguez-Osorio N, Kaya A, Wang H, Page G, Ostermeier G.C, Topper E.K, Memili E (2010). Transcriptome analysis of bull spermatozoa:Implications for male fertility. Reprod. Biomed. Online.

[24] Yathish H.M, Kumar S, Chaudhary R, Mishra C.A.S, Kumar A, Chauchan A, Ghosh S.K, Mitra A (2018). Nucleotide variability of protamine genes influencing bull sperm motility variables. Anim. Reprod. Sci.

[25] Sundararaman M.N, Kalatharan J, Jawahar K.T.P (2012). Computer-assisted semen analysis for quantification of motion characteristics of bull sperm during cryopreservation cycle. Vet. World.

[26] Pardede B.P, Supriatna I, Yudi Y, Agil M (2020). Relationship of frozen-thawed semen quality with the fertility rate after being distributed in the Brahman Cross Breeding Program. Vet. World.

[27] Esteves S.C, Zini A, Coward R.M, Evenson D.P, Gosalvez J, Lewis S.E.M, Sharma R, Humaidan P (2020). Sperm DNA fragmentation testing:Summary evidence and clinical practice recommendations. Andrologia.

[28] Helmi T.Z, Hambal M, Sugito S, Zmzami R.S, Rusli R, Akmal M (2020). Identification and Characterization of Protamine1 Gene in Aceh Cattle. E3S. Web. Conf.

[29] Ismaya (2014). Bioteknologi Inseminasi Buatan Pada Sapi dan Kerbau.

[30] Suyadi S, Herwijanti E, Septian W.A, Furqon A, Nugroho C.D, Putri R.F, Novianti I (2020). Some factors affecting the semen production continuity of elite bulls:reviewing data at Singosari national artificial insemination center (SNAIC), Indonesia. IOP Conf. Earth Environ. Sci.

[31] Mengual L, Ballesca J.L, Ascaso C, Oliva R (2003). Marked differences in protamine content and P1/P2 rations in sperm cells from percoll fractions between patients and control. J. Androl.

[32] Pool K.R, Rickard J.P, de Graaf S.P (2020). Global methylation and protamine deﬁciency in ram spermatozoa correlate with sperm production and quality but are not inﬂuenced by melatonin or season. Animals.

[33] Grzmil P, Boinska D, Kleene K.C, Adham I, Schlüter G, Kämper M, Buyandelger B, Meinhardt A, Wolf S, Engel W (2008). PRM3, the fourth gene in the mouse protamine gene cluster, encodes a conserved acidic protein that affects sperm motility. Biol. Reprod.

[34] Kumar S, Singh U, Ganguly I, Deb R, Singh R, Mann S, Senger G, Mandal D.K, Kumar M, Sharma A (2014). Protamine 3 expressions in crossbred bull spermatozoa may not be a prognostic marker for differentiating good and poor-quality semen. Afr. J. Biotech.

[35] Lv Y.Q, Chen X, Xu D, Luo X.T, Cheng M.M, Zhang Y.Y, Qu X.L, Jin Y (2020). Effects of crocin on frozen-thawed sperm apoptosis, protamine expression and membrane lipid oxidation in Yanbian yellow cattle. Reprod. Domest. Anim.

[36] Vihinen M (2015). Types and effects of protein variations. Hum. Genet.

[37] Pardede B.P, Supriatna I, Yudi Y, Agil M (2020). Decreased bull fertility:Age-related changes in sperm motility and DNA fragmentation. E3S Web Conf.

[38] Badan Standarisasi Nasional (2017). SNI (Standar Nasional Indonesia) Semen Beku Bagian 1 Sapi. Badan Standarisasi Nasional, Jakarta, ID.

[39] Garner D.L, Hafez E.S.E, Hafez B, Hafez E.S.E (2000). Spermatozoa and seminal plasma. Reproduction in Farm Animals.

[40] Kutchy N.A, Menezes E.S.B, Ugur M.R, Husna A.U.I, Eldebaky H, Evans H.C, Beaty E, Santos F.C, Tan W, Wills R.W, Topper E, Kaya A, Moura A.A, Memili E (2019). Sperm cellular and nuclear dynamics associated with bull fertility. Anim. Reprod. Sci.

[41] Miyagawa Y, Nishimura H, Tsujimura A, Matsuoka Y, Matsumiya K, Okuyama A, Nishimune Y, Tanaka H (2005). Single nucleotide polymorphisms and mutation analyses of the TNP1 and TNP2 genes of fertile and infertile human male populations. J. Androl.

[42] Rodríguez-Martínez H (2000). Evaluación de Semen Congelado. Métodos Tradicionales y de Actualidad.

[43] Ohtsuki K, Nishikawa Y, Saito H, Munakata H, Kato T (1996). DNA-binding sperm proteins with oligo-arginine clusters function as potent activators for egg CK-II. FEBS Lett.

[44] Rooney A.P, Zhang J (1999). Rapid evolution of a primate sperm protein:Relaxation of functional constraint or positive Darwinian selection?. Mol. Biol. Evol.

[45] Banaszewska D, Andraszek K, Biesiada-Drzazga B (2015). Evaluation of sperm chromatin structure in boar semen. Bull. Vet. Inst. Pulawy.

[46] Abdillah D.A, Setyawan E.M.N, Oh H.J, Ra K, Lee S.H, Kim M.J, Lee B.C (2019). Iodixanol supplementation during sperm cryopreservation improves protamine level and reduces reactive oxygen species of canine sperm. J. Vet. Sci.

[47] Carreira J.T, Trevizan J.T, Kipper B.H, Perri S.H.V, Carvalho I.R, Rodrigues L.H, Silva C, Koivisto M.B (2015). Impaired protamination and sperm DNA damage in a Nellore bull with high percentages of morphological sperm defects in comparison to normospermic bulls. Arq. Bras. Med. Vet. Zootec.

[48] Borges E, Zanetti B.F, Setti A.S, Braga D.P.A.F, Provenza R.R, Iaconelli A (2019). Sperm DNA fragmentation is correlated with poor embryo development, lower implantation rate, and higher miscarriage rate in reproductive cycles of non-male factor infertility. Fertil. Steril.

[49] Dutta S, Henkel R, Agarwal A (2020). Comparative analysis of tests used to assess sperm chromatin integrity and DNA fragmentation. Anrologia.

[50] Bochenek M, Smorag Z, Pilch J (2001). Sperm chromatin structure assay of bulls qualified for artificial insemination. Theriogenology.

